# From Ovarian to Endocrine-Metabolic Roots: Aligning PCOS Nomenclature with Pathophysiology

**DOI:** 10.1007/s13224-025-02207-4

**Published:** 2025-10-09

**Authors:** Vittorio Unfer

**Affiliations:** 1 The Experts Group on Inositol in Basic and Clinical Research, and on PCOS (EGOI-PCOS), Rome, Italy; 2 UniCamillus-Saint Camillus International University of Health Sciences, Rome, Italy

We read with great appreciation the article “Two decades after Rotterdam consensus: a proposed novel evidence-based practical modifications”, authored by Dr. Mohammad Emam, and published in your esteemed journal [1].

As the Experts Group on Inositol in Basic and Clinical Research, and on PCOS (EGOI-PCOS), we express our strong agreement with the key concepts presented in the paper, which reflect a necessary and long-overdue evolution in the diagnostic and pathogenic framework of polycystic ovary syndrome (PCOS).

We wholeheartedly support the author’s proposal to re-center the pathophysiological interpretation of PCOS around insulin resistance [2]. This paradigm shift—from the long-standing emphasis on obesity as a primary causative factor to recognizing it as an amplifier of pre-existing endocrine-metabolic dysfunction—is not only scientifically sound but also clinically transformative [3]. It compels clinicians and researchers to reconsider the true underlying mechanisms of the syndrome and adopt more tailored, effective therapeutic strategies [4].

Moreover, the paper rightly underscores the heterogeneity of PCOS phenotypes and the importance of stratifying patients not only based on reproductive features but also on metabolic and endocrine profiles. This vision closely mirrors the direction that EGOI-PCOS has advocated in recent years, with a growing international consensus. Notably, we have stressed the importance of redefining the syndrome diagnostic framework to better encompass its core endocrine-metabolic dysfunctions [5].

Notably, just two years after the publication of the ESHRE 2023 international guidelines on PCOS diagnosis and management, ESHRE is reopening the discussion, having recently launched a survey on whether the name “PCOS” should be changed, which culminated with the publication “*Polycystic ovary syndrome perspectives from patients and health professionals on clinical features, current name, and renaming: a longitudinal international online survey*” [6].

This paper revealed that patients and health professionals consider endocrine-metabolic abnormalities—such as insulin resistance, hyperinsulinemia, and metabolic dysfunction—central features of PCOS, often more impactful than ovarian morphology. Importantly, the study highlights how the current name “Polycystic Ovary Syndrome” fails to reflect the core pathophysiology and clinical burden of the condition, particularly its systemic and metabolic components. Many respondents support a change in terminology, arguing for a more accurate name that better captures the endocrine-metabolic nature of the syndrome.

We welcome this development, as it aligns with what EGOI-PCOS has consistently proposed. The term “PCOS” has become inadequate to describe the complex nature of the disorder and fails to capture the nuances of its various aspects.

Our group strongly supports adopting a more pathophysiologically meaningful nomenclature, which could lead to improved diagnostic clarity, better patient communication, and more precise clinical management. Specifically, we advocate for:

- The recognition of the hyperandrogenic and metabolically altered phenotypes as part of an endocrine-metabolic syndrome (EMS), reflecting the systemic involvement of insulin resistance, compensatory hyperinsulinemia, and androgen excess.
- The reclassification of phenotype D—characterized by ovulatory dysfunction and multifollicular ovarian morphology but lacking hyperandrogenism and metabolic derangement—as a multifollicular ovarian disorder (MFOD). In our view, this phenotype does not share the same pathophysiological substrate as the others and should not be grouped under the same diagnostic umbrella.

We are extremely pleased to see these positions echoed and expanded upon in Dr. Emam’s contribution. Articles such as this challenge entrenched paradigms and stimulate much-needed international debate toward a more modern, integrated, and evidence-based conceptualization of PCOS.

Overall, we commend the author for this thoughtful, timely article and we hope that the broader endocrinological and gynecological communities will engage in this important discussion. As EGOI-PCOS, we remain committed to support all scientific efforts aimed at improving the classification, diagnosis, and management of PCOS in ways that better serve both clinical practice and the needs of patients.
